# Fauna of Cerambycidae (Insecta: Coleoptera) in Komaba Campus of the University of Tokyo, a highly urbanised area in Japan

**DOI:** 10.3897/BDJ.5.e22296

**Published:** 2017-12-29

**Authors:** Keiko Kishimoto-Yamada, Junsuke Yamasako, Toshihide Kato, Masayuki U Saito, Motomi Ito

**Affiliations:** 1 Center for Toki and Ecological Restoration, Niigata University, Sado, Niigata, Japan; 2 Department of General Systems Studies, Graduate School of Arts and Science, the University of Tokyo, Meguro, Tokyo, Japan; 3 Faculty of Agriculture, Yamagata University, Tsuruoka, Yamagata, Japan

**Keywords:** arthropods, biodiversity information, herbivore insects, longhorn beetles, urban green space

## Abstract

Urban green spaces play an important role in maintaining urban biodiversity in the Tokyo Metropolis, Japan. Plant-dependent insect assemblages such as Cerambycidae, in particular, are likely influenced by the existence of green spaces in Tokyo’s urbanised environments. This study is the first comprehensive inventory of the cerambycid fauna in the Komaba Campus of the University of Tokyo. A cerambycid assemblage composed of a total of 25 species was recorded within the Komaba Campus site and compared to cerambycid assemblages in nine other green spaces distributed throughout Tokyo. The results indicated that the species number in the campus was similar to that recoded in a similar-sized green space in coastal Tokyo. Fewer cerambycid species were, however, found at the campus site than in larger-sized green spaces within Tokyo. Moreover, species compositions in urbanised areas were markedly different from those in suburbanised parks, mountains and forests within Tokyo.

## Introduction

Urban green spaces, consisting of small patches of forests and grass-lands, play multiple important roles such as providing habitat for plants and animals, recreational areas for humans and improving the local climate (e.g. Mazza and Rydin 1997, Sandström et al. 2006). The central part of the Tokyo Metropolis is highly urbanised, containing patches of relatively large green spaces such as the Imperial Palace (115 ha) and Meiji Jingu (Shinto Shrine, 70 ha). Comprehensive surveys over a 5-year period have been performed in the green spaces of the Imperial Palace and have documented an extremely rich variety of fauna and flora at that location (Takeda et al. 2000, Koyama et al. 2000). For example, 738 coleopteran species belonging to 73 families were recorded at the Imperial Palace site (Nomura et al. 2000). Species diversity was high even in a relatively small green space located within the 25.4 ha Komaba Campus of the University of Tokyo; in total, 115 species (including one newly discovered species) belonging to 29 families of the suborder Heteroptera, were observed at this campus site (Yasunaga et al. 2013, Ishikawa et al. 2015). These findings suggest that urban green spaces play an important role in promoting and maintaining biodiversity in the urban green spaces of the Tokyo Metropolis.

The mountains and forests that surround the central district of Tokyo are also rich in insect species diversity. For example, Mt. Takao (approximately 600 m altitude) has 173 species of Cerambycidae, which is a relatively high number of cerambycid species (comprising approximately 67% of the total cerambycid species number recorded in Tokyo) for a mountain location (Fujita 1988). Suburbanised environments (such as Mt. Takao) may thus contribute to increasing the total number of insect species within the Tokyo Metropolis and may also play an important role in providing source populations of plants and animals to urban green spaces.

Cerambycidae are wood-dependent and their larvae almost exclusively feed on living, dying or dead trees. Approximately 960 species/subspecies of Cerambycidae have been recorded in Japan and, of these, 31% (257 species) live within the administrative districts of Tokyo with a range of habitats from the coastal plains to mountainous regions, excluding islands belonging to the metropolis (Insect Catalogue of Tokyo, Japan, http://tkm.na.coocan.jp, accessed in June 2016). To date, faunal investigations of relatively large-sized urban green spaces have recorded 39 species at the Imperial Palace site (Saito 2000) and 37 species at Meiji Jingu (Okada et al. 2013). Little is known about the fauna of relatively small green spaces on university campuses, or in parks and/or gardens. Knowledge of the biodiversity of such small green spaces will contribute to our overall understanding of urban biodiversity within the Tokyo Metropolis and also promote our understanding of the interactions between Tokyo’s urbanised and suburbanised environments.

The Komaba Campus of the University of Tokyo contains a relatively small green space surrounded by a highly urbanised area of central Tokyo. Appropriately maintained forests, shrubs and grasslands fill spaces amongst a number of buildings and athletic fields within the campus. Two remarkable true bug species (Miridae, Heteroptera) were found recently, living within broadleaf angiosperms on this campus; one was reported as a new species (Yasunaga et al. 2013) and the other as a species that was rediscovered after remaining undetected for 59 years (Ishikawa et al. 2014). These findings provide the basis for future insect inventory surveys for elucidating the biodiversity of urban green spaces.

This study presents the first comprehensive inventory for Cerambycidae in the Komaba Campus and represents an example of extensive research on the fauna of small green spaces within central Tokyo. The study also compares the characteristics of the cerambycid fauna at the campus with those inhabiting urbanised or suburbanised locations within Tokyo.

## Data resources

In total, 61 individuals of 25 species of Cerambycidae were recorded at the Komaba Campus (Suppl. material 1). Of these individuals, 27 individuals were captured by net sweeping, 18 individuals were captured by direct observation during daytime and night-time and 1 individual was sampled by insect fogging. Nine individuals were sampled after emerging from the host woods. For one individual, the sampling method was unknown. Two individuals of *Bacchisa fortunei japonica* (Gahan, 1901) and one individual of *Chlorophorus quinquefasciatus* (Castelnau et Gory, 1841) were included in this paper, although they were captured around the campus. One individual each of *Pseudaeolesthes chrysothrix chrysothrix* (Bates, 1873) and *Nothorhina punctata* (Fabricius, 1798) were observed on campus.

According to the Red List of the administrative districts of Tokyo, published by the Tokyo Metropolitan Government in 2010, *Aegosoma sinicum sinicum* White, 1853 (Fig. 2), *Nothorhina punctata* (Fabricius, 1798), *Dinoptera minuta criocerina* (Bates, 1873), *Paranaspia anaspidoides* (Bates, 1873) (Fig. 3), and *Pseudaeolesthes chrysothrix chrysothrix* (Bates, 1873) were determined as NT (Near threatened). Xylotrechus (Xyloclytus) chinensis kurosawai Fujita, 2010 (Fig. 4) was determined as EN (Endangered). *Acalolepta luxuriosa luxuriosa* (Bates, 1873) was determined as VU (Vulnerable).

## Material and methods

### Study site

**Figure 1.**
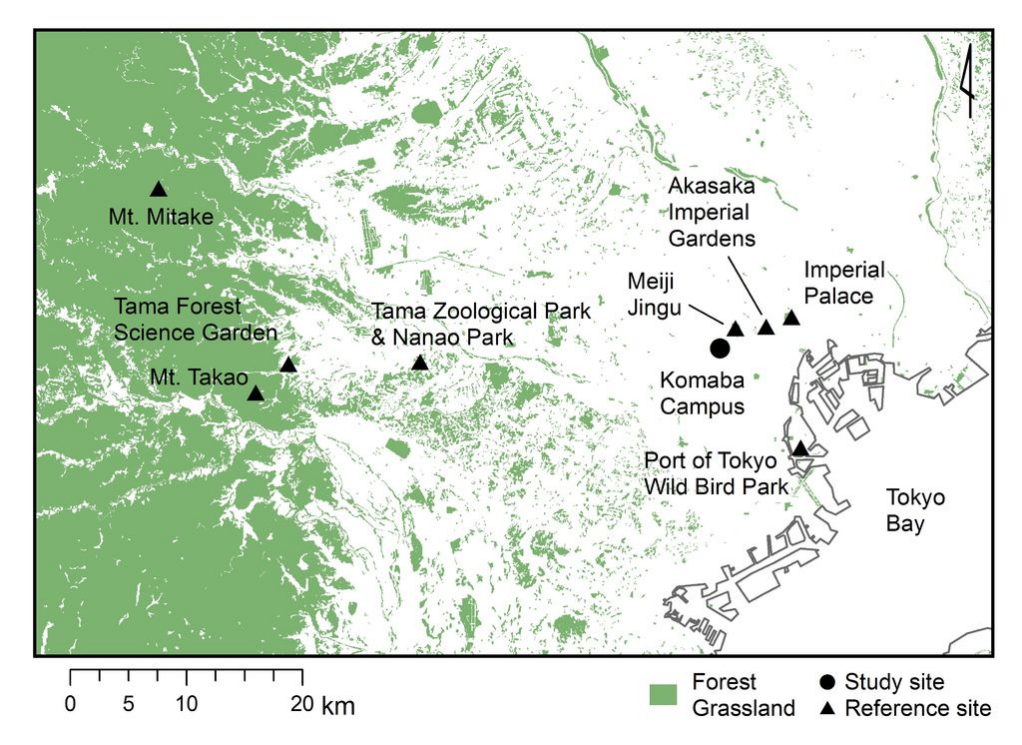
Locations of the Komaba Campus and eight reference sites in Tokyo, Japan.

**Figure 2.**
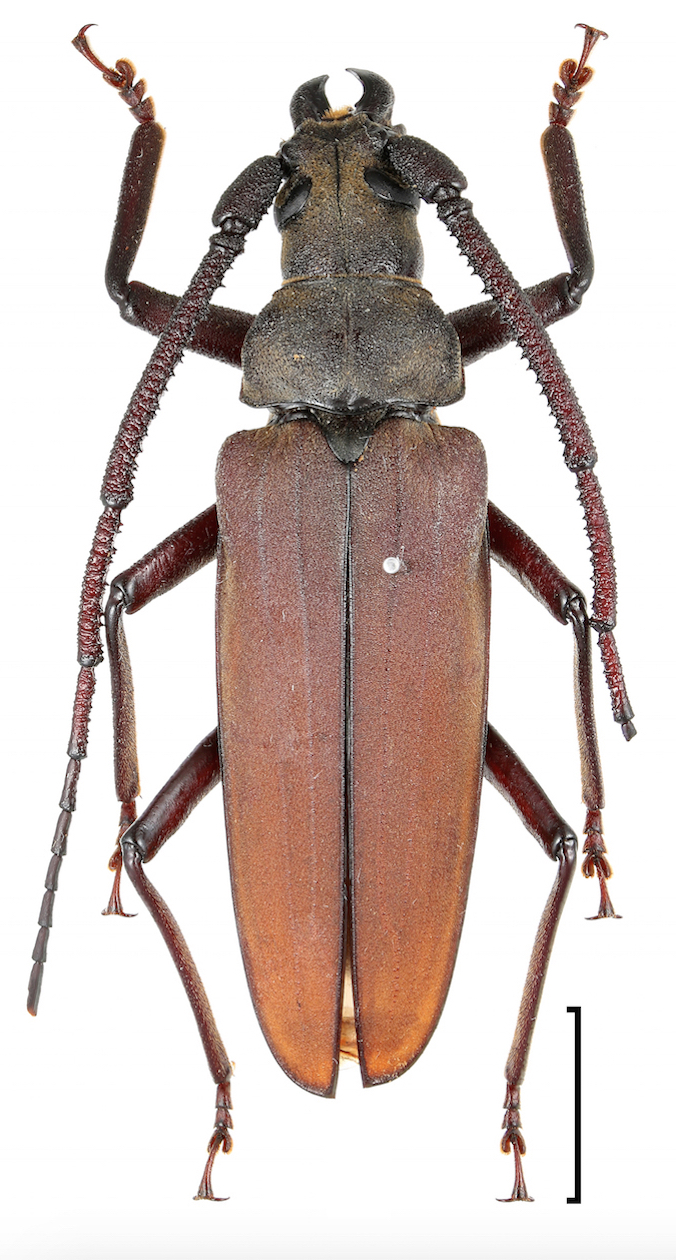
*Aegosoma sinicum sinicum* White, 1853. Scale bar 1 cm.

**Figure 3.**
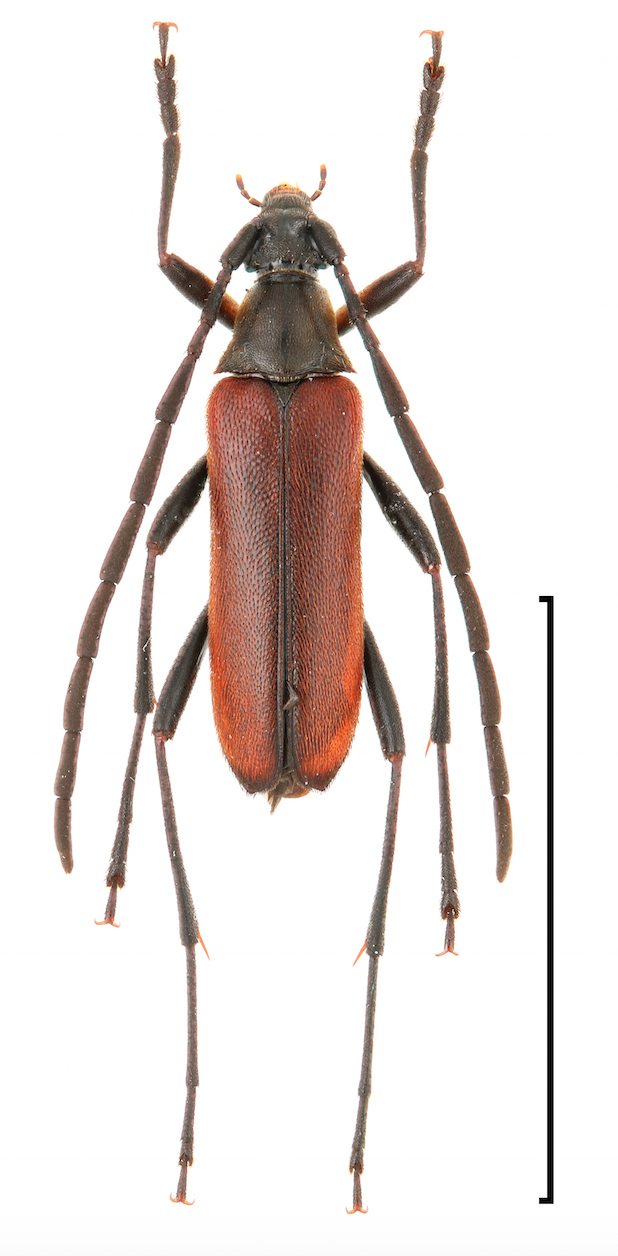
*Paranaspia anaspidoides* (Bates, 1873). Scale bar 1 cm.

**Figure 4.**
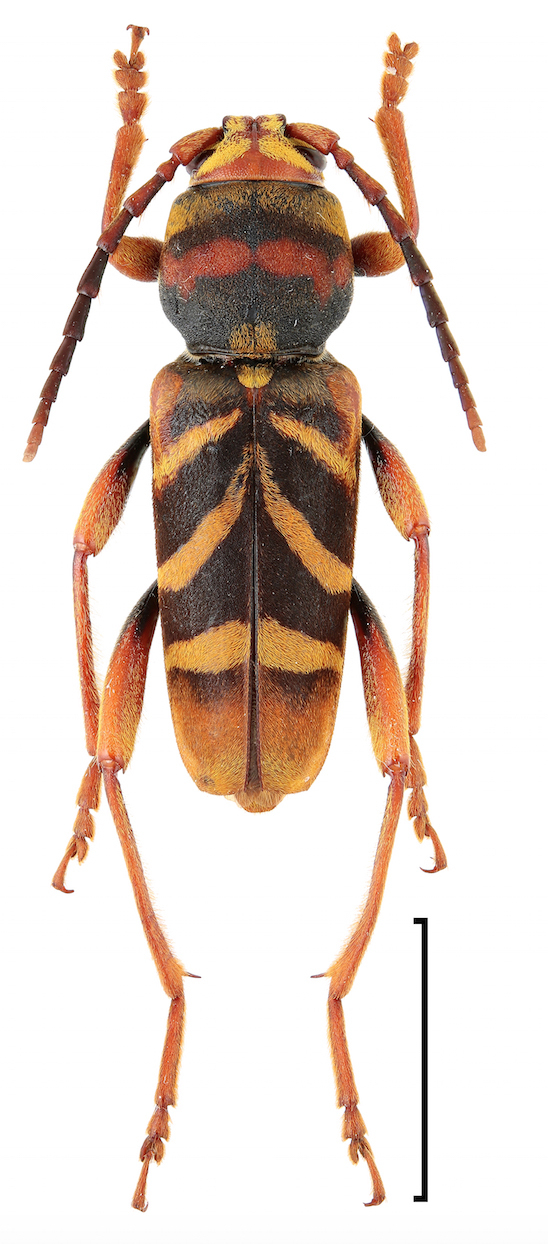
Xylotrechus (Xyloclytus) chinensis kurosawai Fujita, 2010. Scale bar 1 cm.

Specimens were collected in the Komaba Campus (35.66006N, 139.68521E; altitude of approximately 35 m above sea level) of the University of Tokyo, Meguro City, Tokyo, Japan. The campus is situated within central Tokyo and is surrounded by a highly urbanised environment including residential quarters and business complexes (Fig. 1, Ishikawa et al. 2015). The total area of the campus is 25.4 ha, of which 4.5 ha consist of approximately 50 buildings and several athletic fields (http://www.c.u-tokyo.ac.jp/info/about/facts/lands/index.html, accessed on 20th March 2016). The vegetation on campus is characterised by various species of herbs, deciduous/evergreen and broadleaf/coniferous trees. Further details of the study site, including photographs, are provided in Ishikawa et al. (2015). Mean monthly temperatures within Tokyo (1981-2010) varied from 5.2°C (January) to 26.4°C (August), and mean annual precipitation was 1,528.8 mm (Japan Meteorological Agency, accessed in February 2017).

### Sampling methods

Comprehensive sampling of cerambycid fauna was conducted at the study site during May 2014− July 2015, using the following methods: net sweeping, direct observations during daytime and night-time, collecting hosts (collecting and maintaining dead plants until adults emerged from the plants), and insect fogging. Ten specimens were added incidentally sampled on campus in 2004, 2006, 2007, and 2013, to our inventory. *Chlorophorus quinquefasciatus* (Castelnau et Gory, 1841) was further included to the inventory of the campus, even though it was captured outside the campus on 5 August, 2013. This individual was caught within a distance of 40 m from the campus boundary, and was observed (on July 26, 2014) at the campus by one of the authors (T. Kato). Two specimens of Bacchisa (Bacchisa) fortunei japonica (Gahan, 1901) that were found on the leaves of *Photina* plants (Photina × fraseri) outside the campus were also added to the inventory, because the sampling point was within a distance of 20 m from the campus boundary and that *Photina* plants are also found at the campus.

Two additional species were also added to our inventory based on reliable personal observations at the study site; *Pseudaeolesthes chrysothrix chrysothrix* (Bates, 1873) was observed on 13 July, 2013 by T. Kato, and *Nothorhina punctata* (Fabricius, 1798) was observed on 10 September, 2016 by E. Ueda.

All collected specimens were dried at room temperature and mounted for morphological examination. The specimens were preserved in the Insect Collection (IC) at the Komaba Museum, University of Tokyo, Meguro City, Japan (KMUT). Species identification was conducted by J. Yamasako and T. Kato, using Ohbayashi and Niisato (2007).

### Data analysis

The Sørensen similarity index was used based on presence-absence data for biodiversity comparisons between the Komaba Campus and the eight reference sites located outside the campus and distributed throughout Tokyo (Tables 1, 2, Fig. 1). Species compositions were compared amongst sites using cluster analysis with group averaging. These analyses were performed using the ‘Vegan 2.2-1’ package implemented in the R 3.1.2 software environment (R Core Team 2014).

## Results of the data analysis

**Figure 5.**
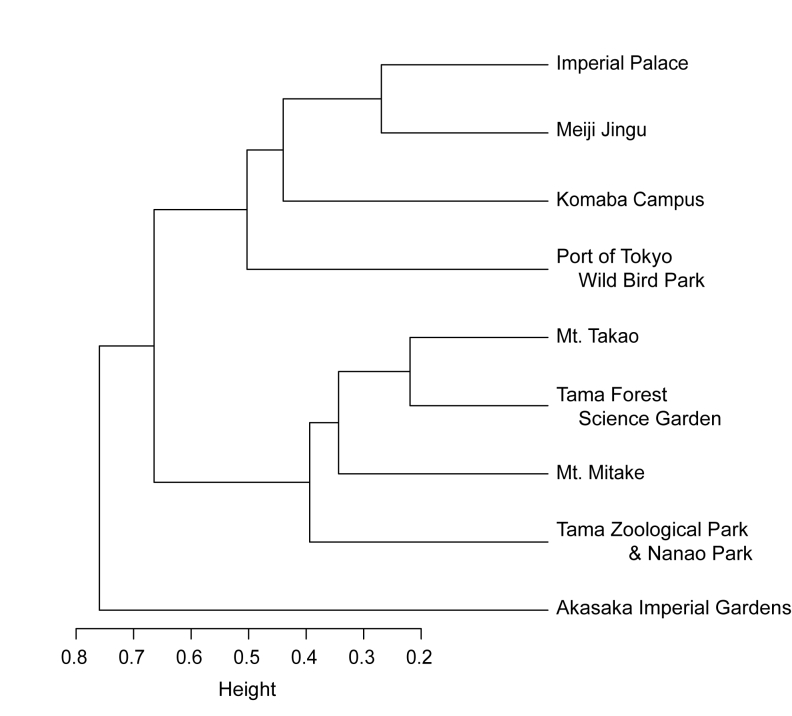
Cluster analysis of Cerambycidae assemblages in the Komaba Campus and eight reference sites, based on the Sørensen similarity index.

Cluster analysis based on the Sørensen similarity index revealed two major cerambycid assemblage groups: one belonging to highly urbanised localities and a reclaimed land (Meiji Jingu, Imperial Palace, Port of Tokyo Wild Bird Park, and the Komaba Campus) and the other belonging to suburbanised localities (Tama Zoological Park & Nanami Park, Mt. Mitake, Tama Forest Science Garden of Forestry and Forest Products Institute and Mt. Takao) (Fig. 5). Moreover, the assemblage of the Akasaka Imperial Gardens was distinctly different from the two major assemblage groups.

## Discussion

In total, 25 species of Cerambycidae were recorded at the Komaba Campus of the University of Tokyo. This species number was similar to that observed in the Port of Tokyo Wild Bird Park, an area of approximately the same size as that of the campus (Table 1). Intensive faunal surveys (1 to 2 per month) were conducted in the Wild Bird Park over a 1-year period (Takakuwa et al. 2015). The species number reported in this study comprised approximately 65% of those observed at the two reference sites (The Imperial Palace and Meiji Jingu, Table 1) that are twice the size of the campus. Cerambycid fauna in these reference sites were also documented via intensive surveys (Saito 2000, Okada et al. 2013). These comparisons indicate that this survey, although not performed as frequently or as quantitatively as those at the reference sites, was sufficiently rigorous for developing the cerambycid fauna list for the Komaba Campus. The campus vegetation is generally mosaic and characterised by various species of herbs, deciduous/evergreen and broadleaf/coniferous trees, including dead branches and woods that are left in place. Such environments likely provide adequate habitat and food resources to help maintain cerambycid species in the campus, despite being in the midst of a highly urbanised environment.

Seven species found in the Komaba Campus are included in the Red List of the administrative districts of Tokyo, excluding islands belonging to the metropolis. Of these individuals, *A. sinicum sinicum* were frequently observed on campus. This species depends on dying and/or dead woods. The forest vegetation at the campus provides a constant supply of dead wood that are then left in place over time, providing habitat and food for *A. sinicum sinicum*. Consequently, populations of this species may survive even in small green spaces such as the campus sites, surrounded by a highly urbanised environments.

Cluster analysis of cerambycid assemblages revealed two major groups (Fig. 5); the group associated with the campus site was characterised by fauna of highly urbanised environments, whereas the other group was characterised by fauna of suburbanised environments. It is likely that large-sized green spaces play an important role as a source for populations of small-sized green spaces. Further studies are required on the insect population structure in green spaces to better understand the biodiversity of urban green spaces.

Cluster analysis further indicated that the fauna of the Akasaka Imperial Gardens was markedly different from the fauna of urbanised and suburbanised areas (Fig. 5). Akasaka Imperial Gardens is located between the Imperial Palace and the Meiji Jingu (Fig. 1) and within a distance of 1.5 km from the Imperial Palace. In the Akasaka Imperial Gardens, forests with broadleaf trees and conifers were distributed over one-third of the total area (Owada and Takeda 2005). A rich fauna representing various insect taxa was observed these areas (Takeda et al. 2000, Owada and Takeda 2005) and had similar species numbers. For example, 463 species of Lepidoptera were found in the Akasaka Imperial Gardens, comprising approximately 90% of the species observed at the Imperial Palace (514 spp.). Poor representation of Cerambycidae species in the fauna of the Akasaka Imperial Palace could be attributed to insufficient sampling.

The Summer Olympics will be held in Tokyo in 2020 and construction of infrastructure and new facilities for the Olympics is well under way. It is believed that the inventory developed in this study for the campus and reference sites provides valuable information that contributes to our understanding of the existing baseline biodiversity of urban green spaces in the Tokyo Metropolis.

## Supplementary Material

Supplementary material 1 KOMABA_CERAM data Data type: occurrences
Brief description: Occurrences of Cerambycidae species at the Komaba Campus.
File: oo_175168.xls K. Kishimoto-Yamada, J. Yamasako

## Figures and Tables

**Table 1.** Detailed characteristics of each reference site. All sites are located in Tokyo, Japan (see Fig. 1).

Locality	Site area (ha)	Environmental aspect	Sampling period of the collection	Number of Species	References
Meiji Jingu	70	highly urbanised	2011-2012	37	Okada et al. 2013
Akasaka Imperial Gardens	51	highly urbanised	2002-2004	12	Nomura and Hirano 2005
Imperial Palace	115	highly urbanised	1996-1998	39	Saito 2000
Port of Tokyo Wild Bird Park	25	reclaimed land	2013-2014	25	Takakuwa et al. 2015
Tama Zoological Park & Nanao Park, Hino City	52.2	suburbanised	1963-2008	83	Sakurai et al. 2008
Tama Forest Science Garden of Forestry and Forest Products Institute, Hachioji City	57.1	suburbanised	1949-2008, Most specimens were collected in the 1990' and 2000'	119	Matsumoto et al. 2014
Mt. Mitake, Ome City	-	suburbanised mountain and forests	1988-2006	120	Yagishita 2008
Mt. Takao, Hachioji City	-	suburbanised mountain and forests	1929-1986, Most specimens were collected in the 1960' and 1970'	173	Fujita 1988

**Table 2.** Species list of the study site and eight reference locations. See references of the data on the presence (+) or absence (-) of species in Table 1. a) Tama Forest Science Garden of Forestry and Forest Products Institute. b) The family Disteniidae is a group within longhorn beetles (i.e. Cerambycidae *s. l.*).

Species	Komaba Campus	Meiji Jingu	Imperial Palace	Akasaka Imperial Gardens	Port of Tokyo Wild Bird Park	Tama Zoological Park & Nanao Park	Tama Forest Science Garden ^a^	Mt. Mitake	Mt. Takao
Disteniidae ^b^									
Disteniinae									
*Distenia gracilis gracilis* (Blessig, 1872)	-	-	+	-	-	+	+	+	+
Cerambycidae									
Prioninae									
*Aegosoma sinicum sinicum* White, 1853	+	+	+	+	-	+	+	+	+
*Prionus insularis insularis* Motschulsky, 1857	-	+	+	-	-	+	+	+	+
*Prionus sejunctus* Hayashi, 1959	-	-	-	-	-	+	+	+	+
*Psephactus remiger remiger* Harold, 1879	-	-	-	-	-	-	+	+	+
Spondylidinae									
*Spondylis buprestoides* (Linnaeus, 1758)	-	-	+	+	-	+	+	+	+
*Nothorhina punctata* (Fabricius, 1798)	+	+	+	-	-	+	-	-	-
*Arhopalus coreanus* (Sharp, 1905)	-	-	-	-	-	+	+	+	+
*Cephalallus unicolor* (Gahan, 1906)	-	-	-	-	-	-	+	+	+
*Megasemum quadricostulatum* Kraatz, 1879	-	-	-	-	-	-	-	+	+
*Asemum striatum* (Linnaeus, 1758)	-	-	-	-	-	-	+	-	+
Lepturinae									
Rhagium (Rhagium) femorale N. Ohbayashi, 1994	-	-	-	-	-	-	+	-	+
Enoploderes (Pyrenoploderes) bicolor Ohbayashi, 1941	-	-	-	-	-	-	-	-	+
*Japanocorus caeruleipennis* (Bates, 1873)	-	-	-	-	-	-	+	+	+
*Toxotinus reinii* (Heyden, 1879)	-	-	-	-	-	-	+	+	+
*Paragaurotes doris* (Bates, 1884)	-	-	-	-	-	-	+	+	-
*Lemula decipiens* Bates, 1884	-	-	-	-	-	-	+	+	+
*Lemula rufithorax* Pic, 1901	-	-	-	-	-	-	-	+	-
*Dinoptera minuta criocerina* (Bates, 1873)	+	+	+	+	-	+	+	+	+
Pidonia (Pseudopidonia) signifera (Bates, 1884)	-	-	-	-	-	-	-	+	-
Pidonia (Pseudopidonia) grallatrix (Bates, 1884)	-	-	-	-	-	-	+	+	+
Pidonia (Mumon) aegrota aegrota (Bates, 1884)	-	-	-	-	-	-	-	+	-
Pidonia (Omphalodera) puziloi (Solsky, 1873)	-	-	-	-	-	-	+	+	+
Pidonia (Cryptopidonia) lyra Kuboki et K. Suzuki, 1978	-	-	-	-	-	-	-	-	+
Pidonia (Cryptopidonia) simillima Ohbayashi et Hayashi, 1960	-	-	-	-	-	-	+	+	-
Pidonia (Cryptopidonia) amentataamentata (Bates, 1884)	-	-	-	-	-	-	+	-	+
*Pseudalosterna misella* (Bates, 1884)	-	-	-	-	-	-	-	+	-
*Kanekoa azumensis* (Matsushita et Tamanuki, 1942)	-	-	-	-	-	-	-	+	-
Anoplodera (Anoploderomorpha) excavata (Bates, 1884)	-	-	-	-	-	-	-	+	+
*Corennys sericata* Bates, 1884	-	-	-	-	-	-	-	+	-
*Judolia japonica* (Tamanuki, 1942)	-	-	-	-	-	-	-	-	+
*Pachytodes cometes* (Bates, 1884)	-	-	-	-	-	-	-	+	-
*Judolidia bangi* (Pic, 1901)	-	-	-	-	-	-	-	+	-
*Anastrangalia scotodes* (Bates, 1873)	-	-	-	-	-	-	+	+	+
Stictoleptura (Aredolpona) succedanea (Lewis, 1879)	-	+	-	-	+	+	+	+	+
Paracorymbia (Batesiata) pyrra (Bates, 1884)	-	-	-	-	-	-	-	+	-
*Konoa granulata* (Bates, 1884)	-	-	-	-	-	-	-	-	+
*Paranaspia anaspidoides* (Bates, 1873)	+	+	+	-	-	+	+	-	+
Leptura (Leptura) dimorpha Bates, 1873	-	-	-	-	-	+	+	+	+
Leptura (Leptura) annularis mimica Bates, 1884	-	-	-	-	-	-	-	-	+
Leptura (Leptura) modicenotata Pic, 1901	-	-	-	-	-	+	+	+	-
Leptura (Leptura) ochraceofasciata ochraceofasciata (Motschulsky, 1861)	-	-	-	-	-	+	+	+	+
Leptura (Leptura) kusamai kusamai K. Ohbayashi et Nakane, 1955	-	-	-	-	-	-	-	+	-
Leptura (Noona) regalis (Bates, 1884)	-	-	-	-	-	-	+	+	+
Pedostrangalia (Neosphenalia) femoralis (Motschulsky, 1860)	-	-	-	-	-	-	-	-	+
*Nakanea vicaria adumbrata* (Bates, 1884)	-	-	-	-	-	-	-	+	-
*Japanostrangalia dentatipennis* (Pic, 1901)	-	-	-	-	-	-	-	+	-
*Strangalia koyaensis* Matsushita, 1933	-	-	-	-	-	-	-	+	-
*Parastrangalis lesnei* (Pic, 1901)	-	-	-	-	-	-	-	+	-
*Parastrangalis tenuicornis* (Motschulsky, 1861)	-	-	-	-	-	-	-	+	+
*Parastrangalis nymphula* (Bates, 1884)	-	-	-	-	-	-	+	+	+
*Idiostrangalia contracta* (Bates, 1884)	-	-	-	-	-	-	+	+	+
*Idiostrangalia hakonensis* (Matsushita, 1933)	-	-	-	-	-	-	-	+	-
*Leptostrangalia hosohana* (Ohbayashi, 1952)	-	-	-	-	-	-	-	+	-
Necydalinae									
Necydalis (Eonecydalis) formosana matsudai Hayashi, 1949	-	-	-	-	-	-	-	+	-
Cerambycinae									
*Neocerambyx raddei* Blessig, 1872	-	-	-	+	-	+	+	-	+
*Pseudaeolesthes chrysothrix chrysothrix* (Bates, 1873)	+	+	+	-	-	+	+	-	+
Margites (Margites) fulvidus (Pascoe, 1858)	-	-	-	-	-	-	-	-	+
*Xystrocera globosa* (Olivier, 1795)	-	-	-	-	-	-	+	-	+
*Trichoferus campestris* (Faldermann, 1835)	-	-	-	-	-	+	-	-	-
Allotraeus (Allotraeus) sphaerioninus Bates, 1877	-	-	-	-	-	-	+	+	+
*Stenygrinum quadrinotatum* Bates, 1873	-	-	-	-	-	+	+	-	+
*Stenodryas clavigera clavigera* Bates, 1873	-	-	-	-	-	-	+	+	+
*Ceresium sinicum* White, 1855	+	+	+	+	+	-	+	-	-
Stenhomalus (Stenhomalus) cleroides Bates, 1873	-	-	-	-	-	+	+	+	+
Stenhomalus (Stenhomalus) taiwanus Matsushita, 1933	-	-	+	-	-	-	-	+	+
Stenhomalus (Stenhomalus) japonicus (Pic, 1904)	-	-	-	-	-	-	-	+	+
Stenhomalus (Stenhomalus) takaosanus Ohbayashi, 1958	-	-	-	-	-	-	-	-	+
*Obrium nakanei* Ohbayashi, 1959	-	-	-	-	-	-	-	-	+
Molorchus (Molorchus) gracilis Hayashi, 1949	-	-	-	-	-	-	+	-	+
Molorchus (Molorchus) kojimai (Matsushita, 1939)	-	+	-	-	-	+	+	+	+
Molorchus (Molorchus) kobotokensis K. Ohbayashi, 1963	-	-	-	-	-	-	-	-	+
*Dere thoracica* White, 1855	-	-	-	-	-	+	+	-	+
*Pyrestes nipponicus* Hayashi, 1987	-	-	-	-	-	-	-	+	+
*Rosalia (Rosalia) batesi* Harold, 1877	-	-	-	-	-	+	+	+	+
*Purpuricenus spectabilis* Motschulsky, 1857	-	-	-	-	-	-	+	+	+
*Purpuricenus temminckii temminckii* (Guérin-Méneville, 1844)	-	-	-	-	-	+	+	+	+
Chloridolum (Parachloridolum) japonicum (Harold, 1879)	-	-	-	-	-	+	-	-	-
Chloridolum (Leontium) viride (Thomson, 1864)	-	-	-	-	-	+	+	-	+
Schwarzerium (Schwarzerium) quadricollis (Bates, 1884)	-	-	-	-	-	+	+	+	+
*Callidiellum rufipenne* (Motschulsky, 1861)	+	-	+	-	-	+	+	+	+
*Semanotus bifasciatus* (Motschulsky, 1875)	-	-	-	-	-	-	-	-	+
*Semanotus japonicus* Lacordaire, 1869	-	-	-	-	-	+	+	-	+
Phymatodes (Phymatodes) testaceus (Linnaeus, 1758)	-	-	-	-	-	+	+	-	+
Phymatodes (Poecilium) maaki viarius Danilevsky, 1988	-	-	-	-	-	-	+	-	+
Phymatodes (Poecilium) quadrimaculatus Gressitt, 1935	-	-	-	-	-	-	-	-	+
Phymatodes (Paraphymatodes) albicinctus (Bates, 1873)	-	-	-	-	-	-	-	-	+
Xylotrechus (Ootora) villioni (Villard, 1892)	-	-	-	-	-	-	-	-	+
Xylotrechus (Xyloclytus) chinensis kurosawai Fujita, 2010	+	-	+	-	+	-	+	-	+
Xylotrechus (Xylotrechus) rufilius rufilius Bates, 1884	-	+	-	-	-	+	+	-	-
Xylotrechus (Xylotrechus) emaciatus Bates, 1884	-	-	-	-	-	-	+	+	+
Xylotrechus (Xylotrechus) pyrrhoderus pyrrhoderus Bates, 1873	-	-	-	-	-	+	+	-	+
Xylotrechus (Xylotrechus) cuneipennis (Kraatz, 1879)	-	-	-	-	-	-	+	+	-
*Brachyclytus singularis* Kraatz, 1879	-	-	-	-	-	-	-	-	+
*Cyrtoclytus caproides caproides* (Bates, 1873)	-	-	-	-	-	+	+	+	+
*Clytus melaenus* Bates, 1884	-	-	-	-	-	-	-	-	+
*Clytus auripilis* Bates, 1884	-	-	-	-	-	-	+	-	+
*Kazuoclytus lautoides* (Hayashi, 1950)	-	-	-	-	-	-	-	-	+
Plagionotus (Plagionotus) christophi (Kraatz, 1879)	-	-	-	-	-	-	-	-	+
*Epiclytus yokoyamai* (Kano, 1933)	-	-	-	-	-	+	-	+	+
*Chlorophorus japonicus* (Chevrolat, 1863)	-	-	-	-	+	+	+	+	+
*Chlorophorus diadema inhirsutus* Matsushita, 1934	-	-	-	-	-	-	-	-	+
*Chlorophorus quinquefasciatus* (Castelnau et Gory, 1841)	+	-	-	-	+	-	-	-	-
*Chlorophorus annularis* (Fabricius, 1787)	-	-	-	-	-	+	-	-	+
*Chlorophorus muscosus* (Bates, 1873)	-	-	-	-	+	-	-	-	-
*Rhaphuma xenisca* (Bates, 1884)	-	-	-	-	-	+	+	+	+
*Rhaphuma diminuta diminuta* (Bates, 1873)	+	+	+	-	+	+	+	-	+
*Grammographus notabilis notabilis* (Pascoe, 1862)	-	+	-	-	-	+	+	+	+
*Demonax transilis* Bates, 1884	-	+	+	-	-	+	+	+	+
*Paraclytus excultus* Bates, 1884	-	-	-	-	-	-	+	+	+
Anaglyptus (Anaglyptus) niponensis Bates, 1884	-	+	+	+	-	-	+	+	+
Anaglyptus (Anaglyptus) matsushitai Hayashi, 1955	-	-	-	-	-	-	-	+	+
Anaglyptus (Akajimatora) bellus bellus Matsumura et Matsushita, 1933	-	-	-	-	-	-	+	+	+
Lamiinae									
Falsomesosella (Falsomesosella) gracilior (Bates, 1884)	-	-	-	-	-	-	+	-	+
Mesosa (Mesosa) mediofasciata Breuning, 1942	-	-	-	-	-	-	+	-	+
Mesosa (Mesosa) japonica Bates, 1873	-	-	-	-	-	+	+	+	+
Mesosa (Perimesosa) hirsuta hirsuta Bates, 1884	+	+	+	-	+	+	+	+	+
Mesosa (Aplocnemia) longipennis Bates, 1873	+	+	+	-	+	+	+	+	+
Mesosa (Aplocnemia) senilis Bates, 1884	-	-	-	-	-	-	-	+	+
*Asaperda agapanthina* Bates, 1873	-	+	+	+	-	+	+	+	+
*Asaperda rufipes* Bates, 1873	-	-	-	-	-	-	+	-	+
*Apomecyna naevia naevia* Bates, 1873	+	+	+	-	+	+	-	-	+
*Atimura japonica* Bates, 1873	-	-	+	-	+	-	-	+	+
*Xylariopsis mimica* Bates, 1884	-	-	-	-	-	-	-	-	+
*Microlera ptinoides* Bates, 1873	-	+	+	-	-	-	+	+	+
Sybra (Sybra) flavomaculata Breuning, 1939	-	-	-	-	-	-	-	-	+
Sybra (Microzotale) kuri Ohbayashi et Hayashi, 1962	-	-	-	-	-	-	-	-	+
Sybra (Sybrodiboma) subfasciata subfasciata Bates, 1884	-	-	-	-	-	-	+	+	+
*Aulaconotus pachypezoides* Thomson, 1864	-	-	-	-	-	-	+	-	+
*Cleptometopus bimaculatus* (Bates, 1873)	-	-	-	-	-	+	-	+	+
*Pseudocalamobius japonicus* (Bates, 1873)	-	-	-	-	-	-	-	+	+
Egesina (Niijimaia) bifasciana bifasciana (Matsushita, 1933)	+	+	-	-	-	+	-	+	+
Pterolophia (Pterolophia) leiopodina leiopodina (Bates, 1873)	-	-	-	-	-	-	-	-	+
Pterolophia (Pterolophia) angusta (Bates, 1873)	-	-	-	-	-	+	+	-	+
Pterolophia (Pterolophia) zonata (Bates, 1873)	+	+	+	+	+	+	+	-	+
Pterolophia (Pterolophia) castaneivora Ohbayashi et Hayashi, 1962	-	-	-	-	-	+	-	+	+
Pterolophia (Pterolophia) tsurugiana (Matsushita, 1934)	-	-	-	-	-	-	-	+	+
Pterolophia (Pterolophia) caudata caudata (Bates, 1873)	-	+	+	-	+	+	+	+	+
Pterolophia (Pterolophia) granulata (Motschulsky, 1866)	+	+	+	-	-	+	+	+	+
Pterolophia (Ale) jugosa jugosa (Bates, 1873)	-	-	-	-	-	+	+	+	+
Pterolophia (Hylobrotus) annulata (Chevrolat, 1845)	-	-	+	-	+	-	+	-	+
*Mesosella simiola* Bates, 1884	-	-	-	-	-	-	-	+	-
Niphona (Niphona) furcata (Bates, 1873)	-	-	-	-	-	+	+	-	+
*Mecynippus pubicornis* Bates, 1884	-	-	-	-	-	+	-	-	+
Monochamus (Monochamus) alternatus endai Makihara, 2004	-	-	-	-	-	+	+	-	+
Monochamus (Monochamus) grandis Waterhouse, 1881	-	-	-	-	-	-	+	+	+
Monochamus (Monochamus) subfasciatus subfasciatus Bates, 1873	-	+	-	-	-	+	+	+	+
*Anoplophora malasiaca* (Thomson, 1865)	-	-	-	-	+	+	+	-	+
*Acalolepta luxuriosa luxuriosa* (Bates, 1873)	+	+	-	-	-	+	+	+	+
*Acalolepta fraudatrix fraudatrix* (Bates, 1873)	-	+	+	-	-	+	+	+	+
*Acalolepta kusamai* Hayashi, 1969	-	-	+	+	-	-	+	-	+
*Acalolepta sejuncta sejuncta* (Bates, 1873)	-	+	+	-	-	-	+	+	+
*Acalolepta degener* (Bates, 1873)	-	-	-	-	-	-	-	-	+
*Uraecha bimaculata bimaculata* Thomson, 1864	-	+	+	+	-	+	+	+	+
*Psacothea hilaris hilaris* (Pascoe, 1857)	+	+	+	-	+	+	+	+	+
*Eupromus ruber* (Dalman, 1817)	-	-	+	-	+	-	-	-	-
*Dolichoprosopus yokoyamai* (Gressitt, 1937)	-	-	-	-	-	-	-	-	+
*Xenicotela pardalina* (Bates, 1884)	-	-	-	-	-	-	+	+	+
*Apriona rugicollis rugicollis* Chevrolat, 1852	-	+	+	-	+	+	+	+	+
*Batocera lineolata* Chevrolat, 1852	-	-	-	-	-	+	+	+	+
*Palimna liturata* (Bates, 1884)	-	-	-	-	-	-	-	-	+
*Rhodopina lewisii lewisii* (Bates, 1873)	-	-	-	-	-	+	+	+	+
*Rhopaloscelis unifasciatus* Blessig, 1873	-	-	-	-	+	+	+	+	+
*Rhopaloscelis maculatus* Bates, 1877	-	-	-	-	-	-	+	+	+
*Arhopaloscelis bifasciata* (Kraatz, 1879)	-	+	+	-	-	-	+	+	+
*Graphidessa venata venata* Bates, 1884	-	-	-	-	-	-	+	+	-
Miccolamia (Miccolamia) cleroides Bates, 1884	-	-	-	-	-	-	-	+	+
Miccolamia (Isomiccolamia) verrucosa Bates, 1884	-	-	-	-	-	-	-	-	+
*Cylindilla grisescens* Bates, 1884	-	-	-	-	-	-	-	+	-
*Mimectatina divaricata divaricata* (Bates, 1884)	-	-	-	-	-	-	+	-	+
*Eupogoniopsis tenuicornis* (Bates, 1884)	-	-	-	-	-	-	-	+	+
*Sophronica obrioides* (Bates, 1873)	-	-	-	-	-	-	-	-	+
Pogonocherus (Pogonocherus) dimidiatus Blessig, 1873	-	-	-	-	-	-	+	-	+
*Callapoecus guttatus* Bates, 1884	-	-	-	-	-	-	-	+	-
Acanthocinus (Acanthocinus) orientalis Ohbayashi, 1939	-	-	-	-	-	+	+	-	+
Acanthocinus (Acanthobatesianus) guttatus (Bates, 1873)	-	-	-	-	-	+	-	-	+
*Leiopus stillatus* (Bates, 1884)	-	-	-	-	-	-	+	-	+
Rondibilis (Rondibilis) saperdina (Bates, 1884)	-	-	-	-	-	+	-	+	+
Rondibilis (Rondibilis) sapporensis (Matsushita, 1933)	-	-	-	-	-	-	-	-	+
Exocentrus (Exocentrus) testudineus Matsushita, 1931	-	-	+	-	-	-	+	+	+
Exocentrus (Exocentrus) galloisi Matsushita, 1933	-	+	+	-	-	+	+	+	+
Exocentrus (Exocentrus) lineatus Bates, 1873	+	+	-	-	+	+	+	-	+
Exocentrus (Exocentrus) fasciolatus Bates, 1873	+	+	+	-	+	+	-	-	+
Exocentrus (Pseudocentrus) guttulatus Bates, 1873	-	-	+	-	+	+	+	-	+
*Miaenia tonsa* (Bates, 1873)	+	-	+	+	+	+	-	+	+
Saperda (Lopezcolonia) tetrastigma Bates, 1879	-	-	-	-	-	-	+	-	+
*Eutetrapha ocelota* (Bates, 1873)	-	+	+	-	+	+	+	+	+
*Pareutetrapha eximia* (Bates, 1884)	-	-	-	-	-	-	+	-	+
*Pareutetrapha simulans* (Bates, 1873)	-	-	-	-	-	-	-	+	+
*Cagosima sanguinolenta* Thomson, 1864	-	-	-	-	-	+	+	-	+
*Thyestilla gebleri* (Faldermann, 1835)	-	-	-	-	-	-	-	-	+
*Paraglenea fortunei* (Saunders, 1853)	+	-	-	-	-	-	+	+	-
*Praolia citrinipes citrinipes* Bates, 1884	-	-	-	-	-	-	+	+	+
Menesia (Menesia) sulphurata (Gebler, 1825)	-	-	-	-	-	-	-	+	-
Menesia (Menesia) flavotecta Heyden, 1886	-	-	-	-	-	-	+	-	+
Glenea (Glenea) centroguttata Fairmaire, 1897	-	-	-	-	-	-	-	-	-
Glenea (Glenea) relicta relicta Pascoe, 1858	-	+	-	-	-	+	+	+	+
*Eumecocera gleneoides* (Gressitt, 1935)	-	-	-	-	-	-	-	-	+
*Eumecocera trivittata* (Breuning, 1947)	-	-	-	-	-	-	+	-	+
Phytoecia (Phytoecia) coeruleomicans Breuning, 1946	-	-	-	-	-	-	-	-	+
Phytoecia (Phytoecia) rufiventris Gautier, 1870	+	-	-	-	-	+	-	-	+
*Epiglenea comes comes* Bates, 1884	-	+	-	-	-	+	+	+	+
*Nupserha marginella* (Bates, 1873)	-	-	-	-	-	+	+	+	+
Oberea (Oberea) hebescens Bates, 1873	-	-	-	-	-	+	+	+	+
Oberea (Oberea) japonica (Thunberg, 1787)	+	-	-	+	-	+	+	+	+
Oberea (Oberea) yasuhikoi Kusakabe, 2001	-	-	-	-	+	-	-	-	-
Oberea (Oberea) sobosana K. Ohbayashi, 1956	-	-	-	-	-	-	-	+	-
Oberea (Oberea) mixta Bates, 1873	-	-	-	-	-	-	-	-	+
Oberea (Oberea) shirahatai K. Ohbayashi, 1956	-	-	-	-	-	+	-	-	-
Oberea (Oberea) infranigrescens Breuning, 1947	-	-	-	-	-	+	-	+	+
Oberea (Oberea) gracillima Pascoe, 1867	-	-	-	-	-	-	-	-	+
Bacchisa (Bacchisa) fortunei japonica (Gahan, 1901)	+	-	-	-	-	+	-	-	+
